# Supplementary material for: The Design, Synthesis and Application of Nitrogen Heteropolycyclic Compounds with UV Resistance Properties
Source: Int J Mol Sci. 2023 Apr 26;24(9):7882. doi: 10.3390/ijms24097882 (PMC10178182; doi:10.3390/ijms24097882)
Supplement: Supplementary file 1 [file ijms-24-07882-s001.zip › ijms-2331884-supplementary.pdf]

Supporting Information

The Design, Synthesis and Application of Nitrogen Heteropolycyclic Compounds with Anti-UV Properties

Biao Yang Error! Bookmark not defined.**, Xinbo Yang** [1,2] **, Yuchuan Li**, [1]*** and Siping Pang** [1,]*

[1]*School of Materials Science and Engineering, Beijing Institute of technology, Beijing, 100081, China*

[2]*School of Mechatronical Engineering, Beijing Institute of Technology, Beijing, 100081, China*

*Correspondence to: Yuchuan Li, and Siping Pang (E-mail: liyuchuan@bit.edu.cn; pangsp@bit.edu.cn)

TABLE OF CONTENTS

1. NMR spectra 3

2. Mass spectra 7

3. Ultraviolet absorption spectrum 9

4. Single crystal X diffraction pattern 10

5. Degradation of the film during aging 15

1. NMR spectra

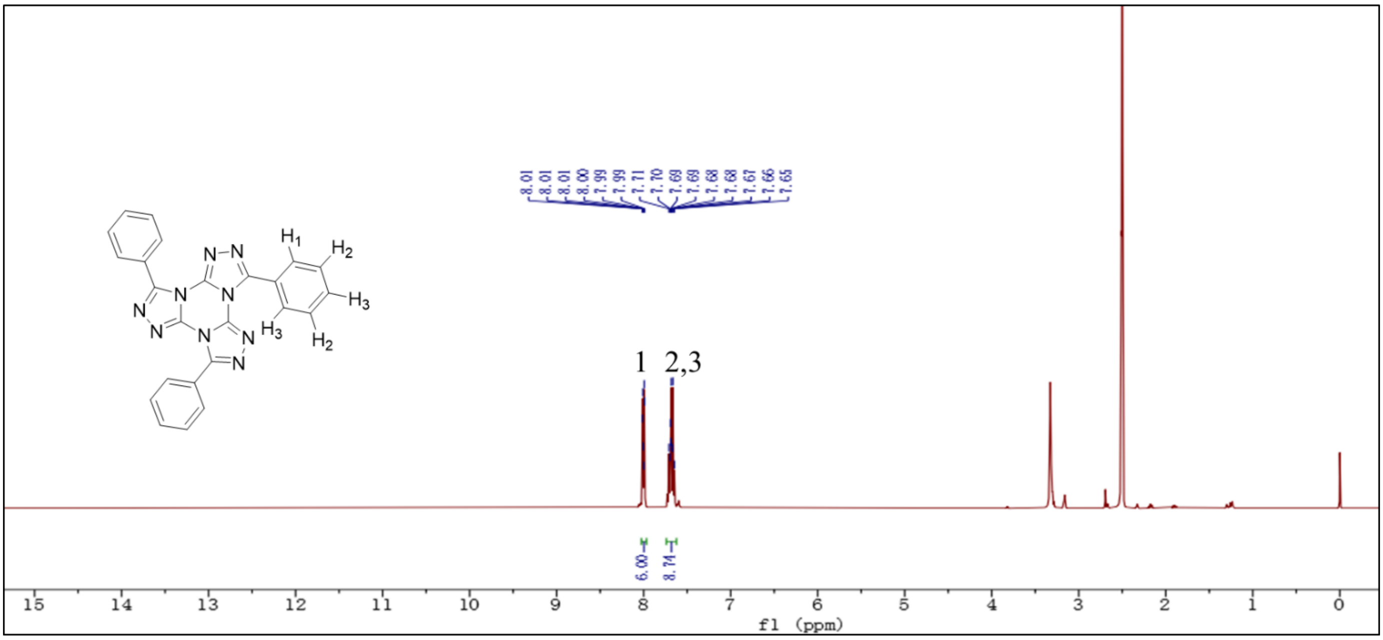

Figure S1. ^{1}H NMR spectrum (400 MHz) in DMSO-d_6 for compound TTTB.

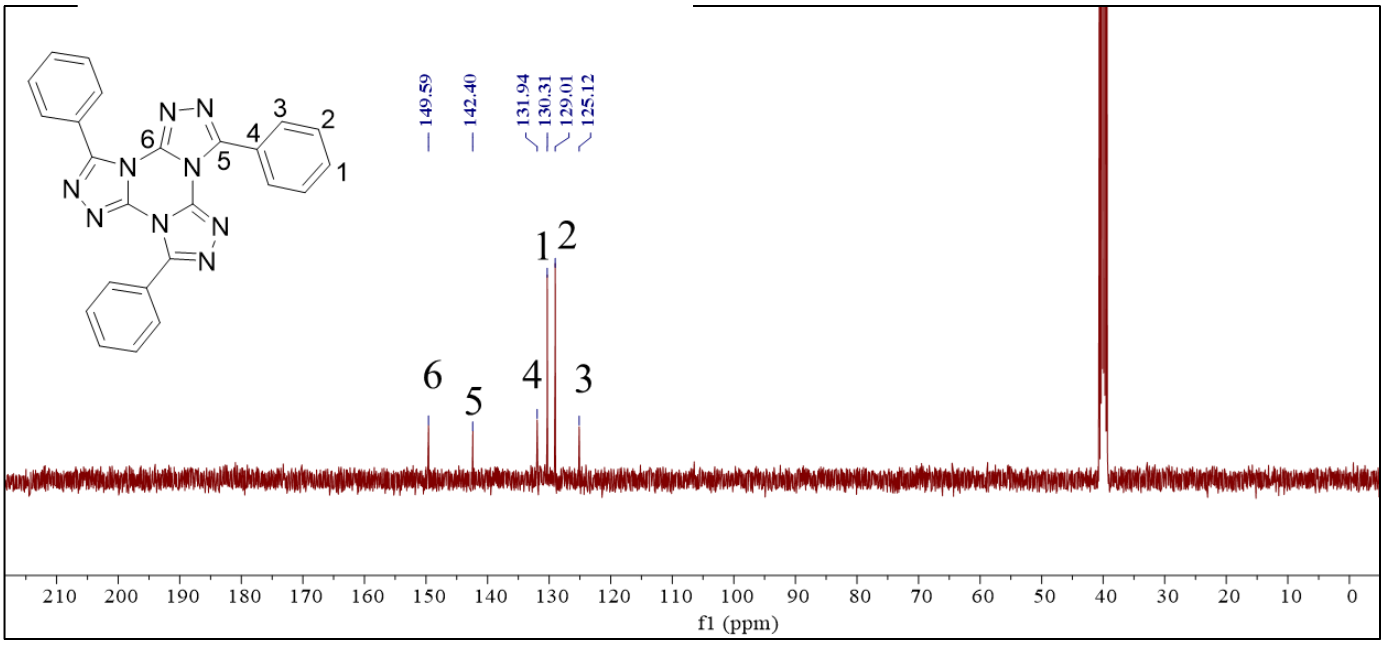

Figure S2. ^{13}C NMR spectrum (100 MHz) in DMSO-d_6 for compound TTTB.

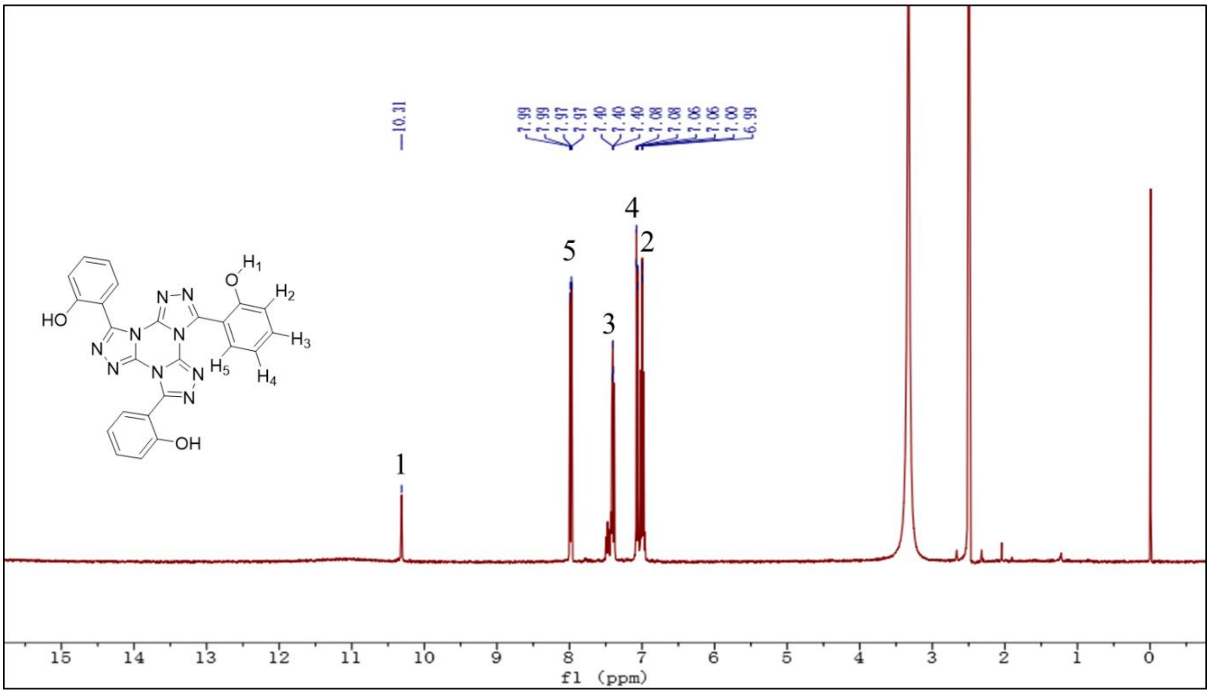

Figure S3. ^{1}H NMR spectrum (400 MHz) in $CDCl_3$ for compound TTTL.

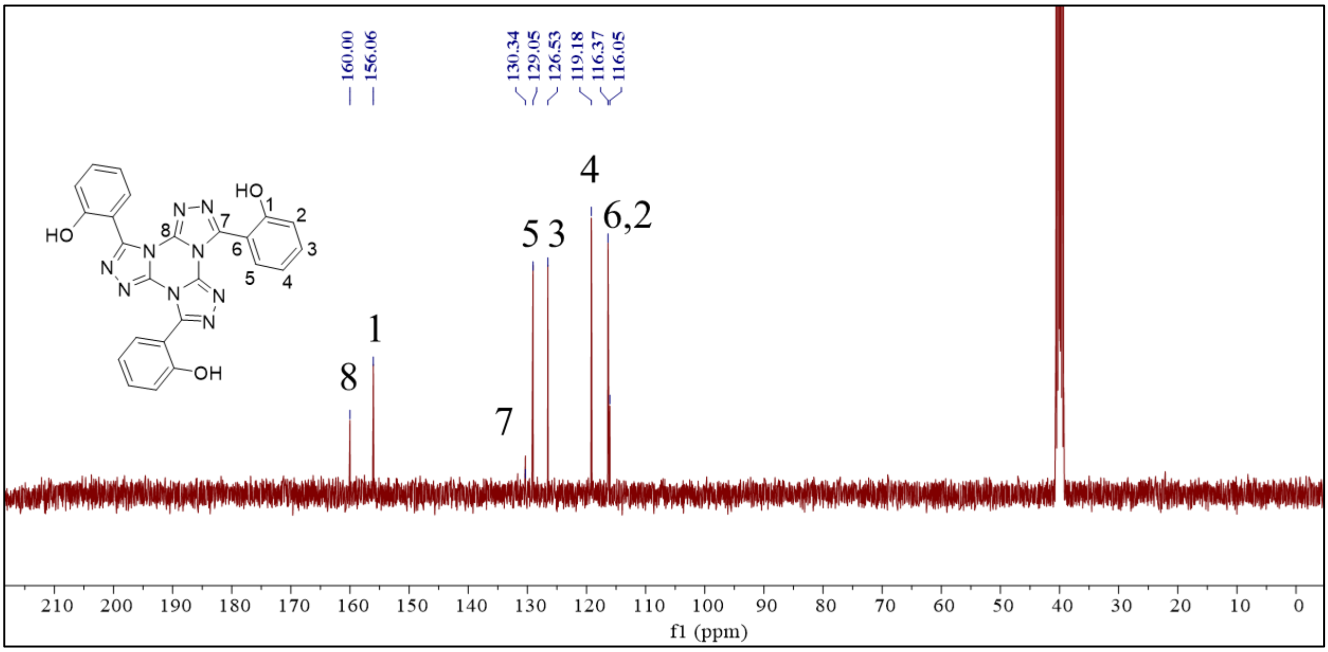

Figure S4. ^{13}C NMR spectrum (100 MHz) in $CDCl_3$ for compound TTTL.

.

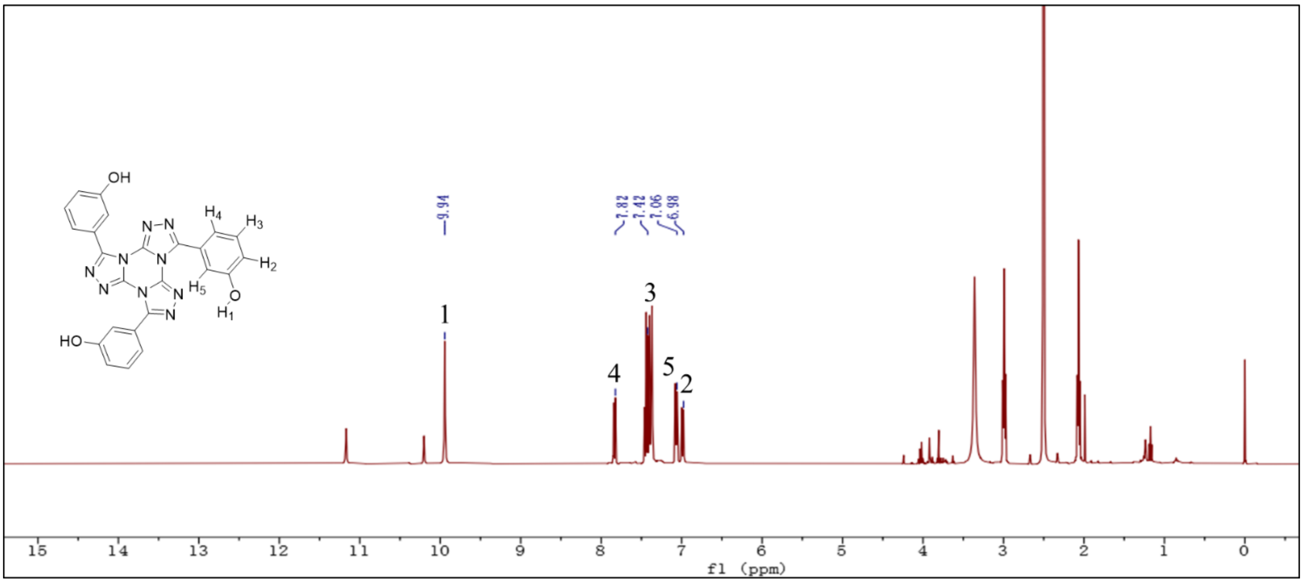

Figure S5. ^{1}H NMR spectrum (400 MHz) in $CDCl_3$ for compound TTTJ.

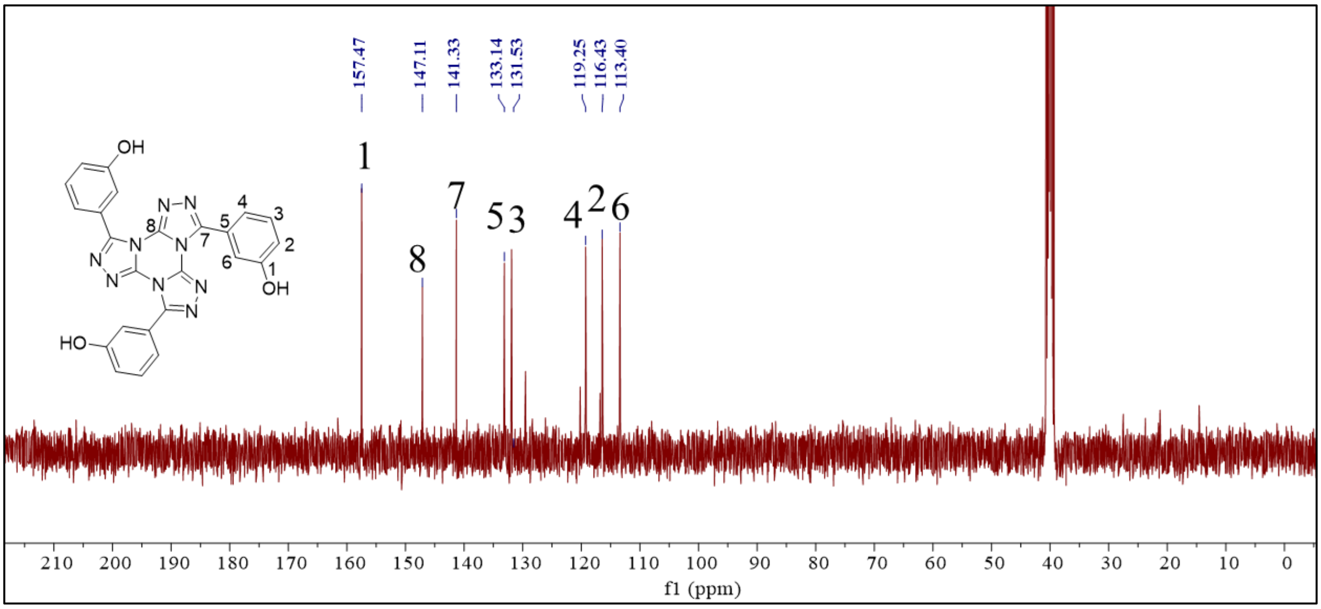

Figure S6. ^{13}C NMR spectrum (100 MHz) in $CDCl_3$ for compound TTTJ.

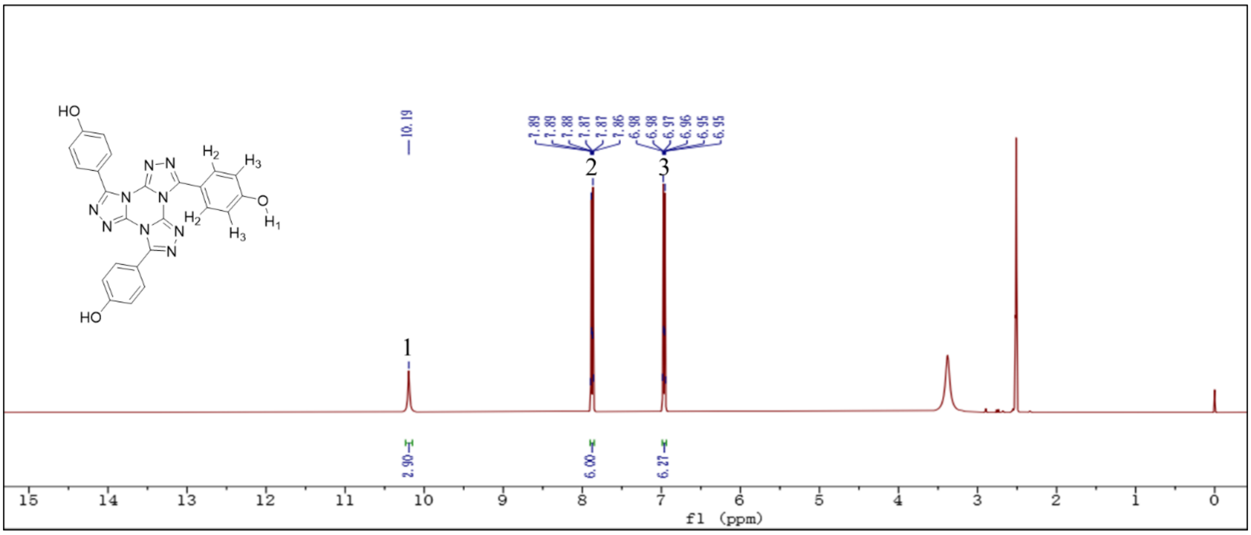

Figure S7. ^{1}H NMR spectrum (400 MHz) in $CDCl_3$ for compound TTTD.

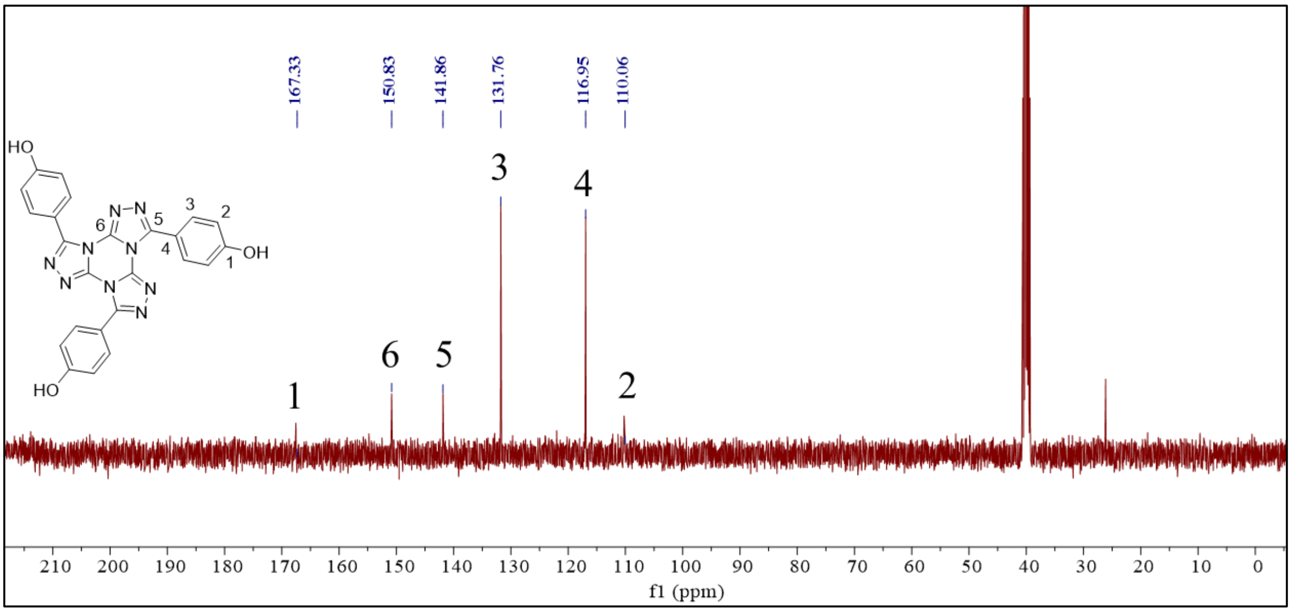

Figure S8. ^{13}C NMR spectrum (100 MHz) in $CDCl_3$ for compound TTTD.

2. Mass spectra

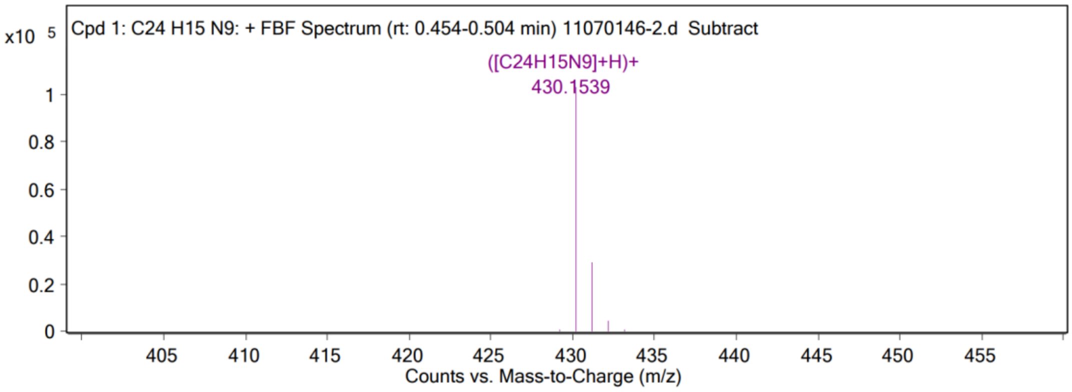

Figure S9. Mass spectrum of compound TTTB.

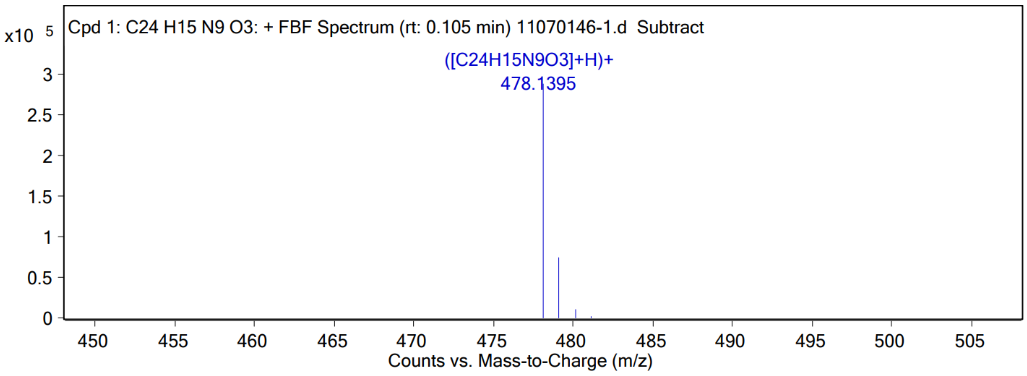

Figure S10. Mass spectrum of compound TTTL.

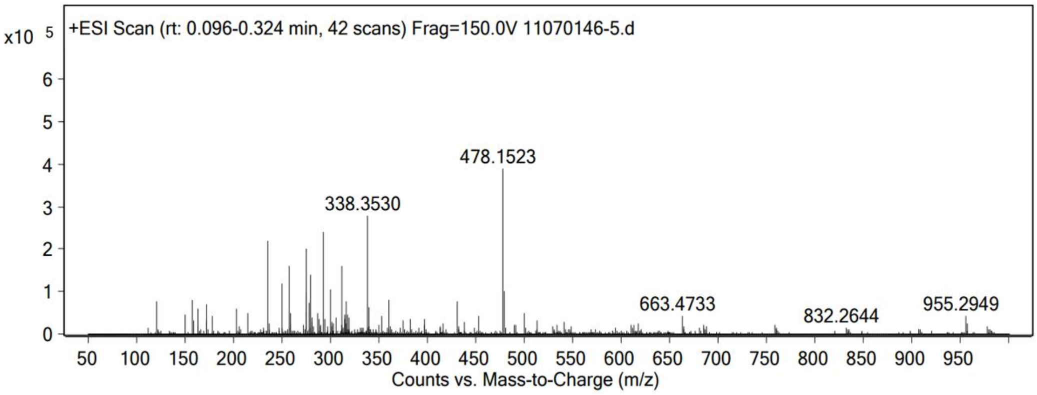

Figure S11. Mass spectrum of compound TTTJ.

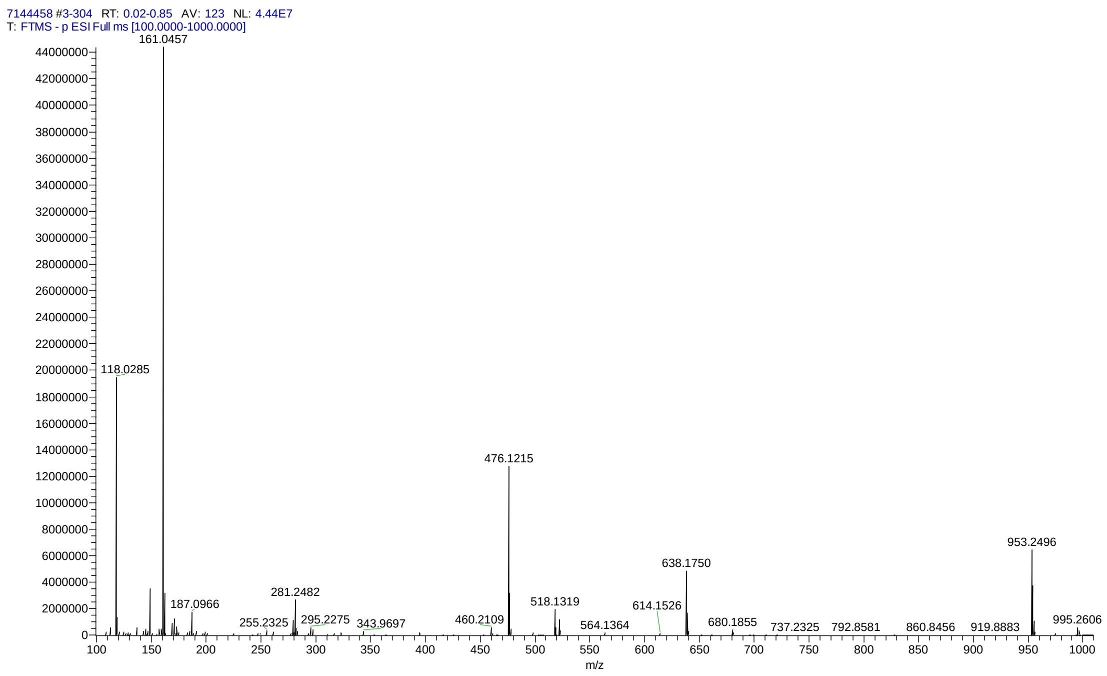

Figure S12. Mass spectrum of compound TTTD.

3. Ultraviolet absorption spectrum

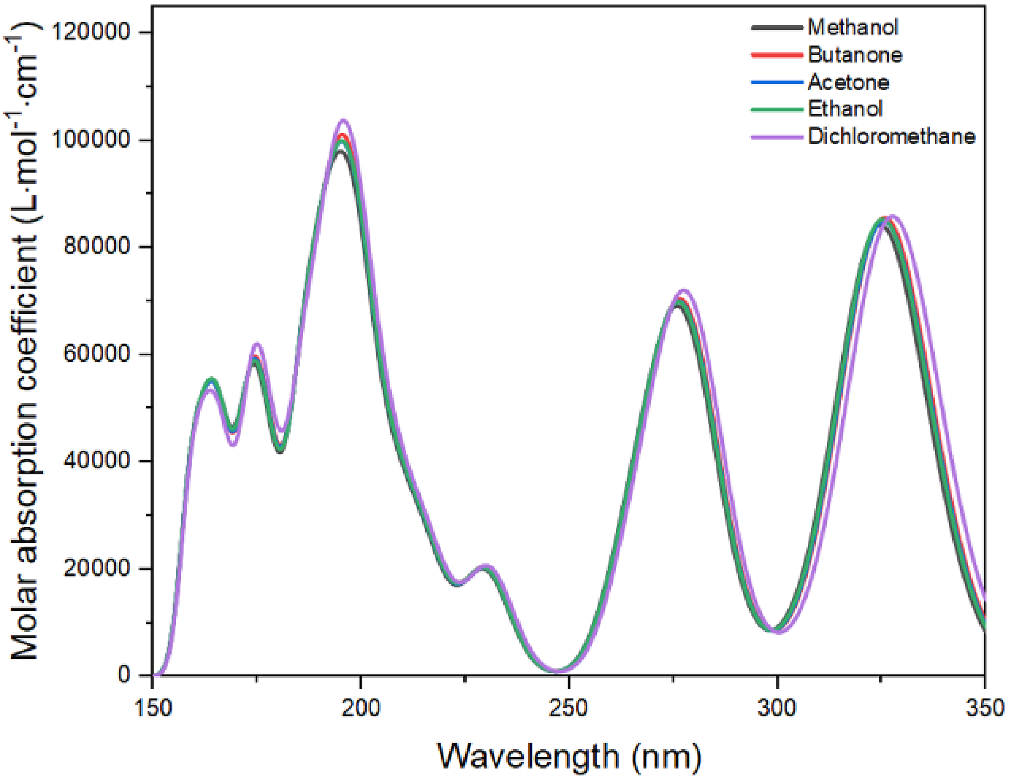

Figure S13.UV-visible absorption spectra of TTTL in different organic solvents were simulated.

4. Single crystal X diffraction pattern

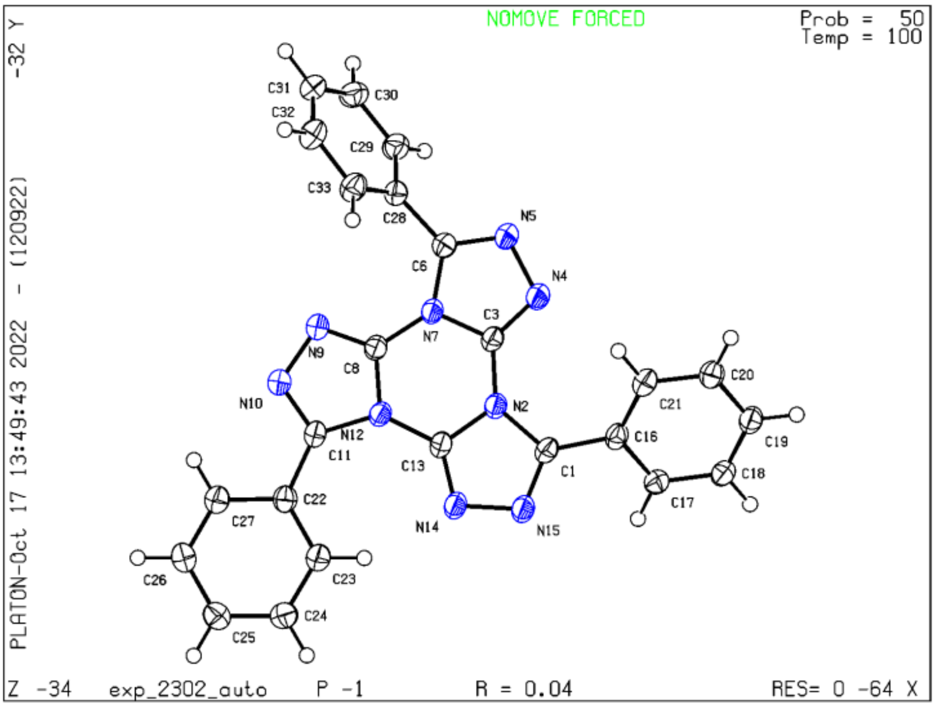

Figure S14. Infrared spectrum of compound TTTB.

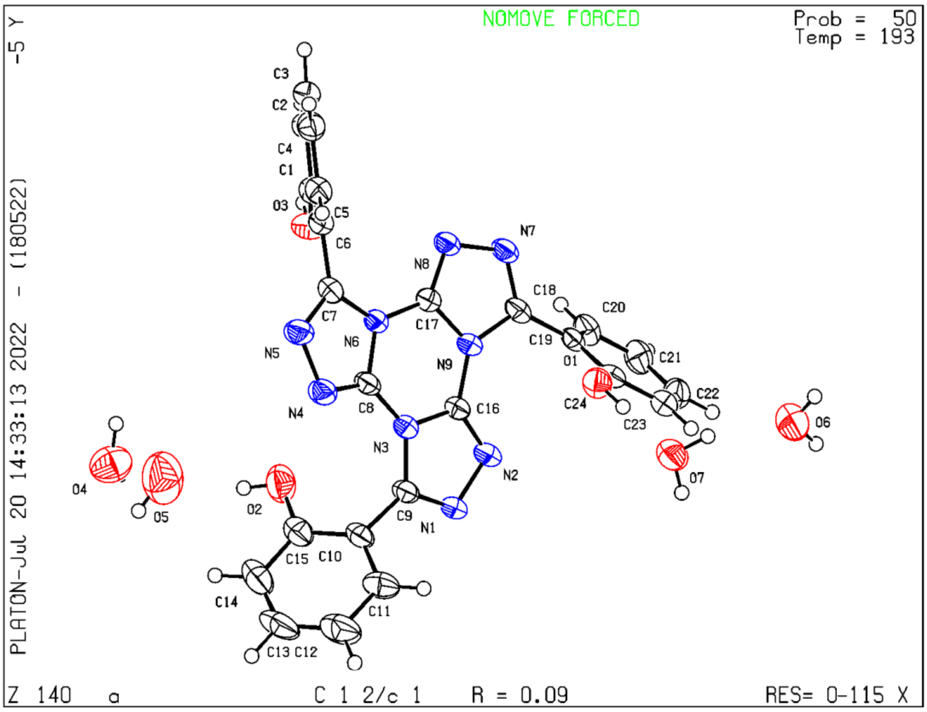

Figure S15. Infrared spectrum of compound TTTL.

Table S1. Bond Lengths for TTTB.

Chemical bond	Length / Å	Chemical bond	Length / Å
N2-C3	1.3899	C16-C1	1.4736
N2-C13	1.3857	C11-C22	1.4767
N2-C1	1.3955	C6-C28	1.4704
N12-C11	1.413	C20-C21	1.3882
N12-C8	1.3775	C20-C19	1.385
N12-C13	1.4022	C22-C23	1.393
N7-C3	1.3706	C22-C27	1.407
N7-C6	1.3905	C17-C18	1.3873
N7-C8	1.3895	C23-C24	1.391
N15-N14	1.3977	C28-C33	1.3936
N15-C1	1.307	C28-C29	1.389
N14-C13	1.2925	C26-C27	1.382
N5-N4	1.4007	C26-C25	1.388
N5-C6	1.3081	C19-C18	1.382
N4-C3	1.2925	C25-C24	1.387
N9-N10	1.3885	C33-C32	1.383
N9-C8	1.2893	C29-C30	1.384
N10-C11	1.3075	C32-C31	1.384
C16-C17	1.3981	C30-C31	1.388
C16-C21	1.393		

Table S2. Bond Lengths for TTTL.

Chemical bond	Length / Å	Chemical bond	Length / Å
O1-C24	1.351	C7-C6	1.46
O3-C1	1.363	C6-C1	1.393
N6-C17	1.394	C6-C5	1.386
N6-C7	1.392	C19-C18	1.472
N6-C8	1.375	C19-C24	1.396
N9-C17	1.37	C19-C20	1.392
N9-C16	1.389	C9-C10	1.468
N9-C18	1.385	C1-C2	1.392
N3-C16	1.369	C24-C23	1.393
N3-C9	1.386	C3-C2	1.376
N3-C8	1.387	C3-C4	1.393
N7-N8	1.408	C10-C15	1.376
N7-C18	1.309	C10-C11	1.4
O2-C15	1.348	C23-C22	1.388
N1-N2	1.396	C20-C21	1.379
N1-C9	1.307	C5-C4	1.382
N8-C17	1.287	C21-C22	1.37
N2-C16	1.291	C15-C14	1.399
N4-N5	1.401	C11-C12	1.369
N4-C8	1.292	C14-C13	1.364
N5-C7	1.311	C13-C12	1.371

Table S3. Crystal data and structure refinement for TTTB.

Empirical formula	$C_{24}H_{15}N_9$
Formula weight	429.45
Temperature/K	100.01
Crystal system	triclinic
Space group	*P-1*
a /Å	6.08034 (15)
b /Å	12.5701 (4)
c /Å	13.7523 (4)
α / °	110.383 (3)
β / °	92.436 (2)
γ / °	102.329 (2)
V /Å^3	954.69 (5)
Z	2
ϱ / (g·cm^{-3})	1.494
μ /mm^{-1}	0.780
Crystal size / mm^3	0.2 × 0.15 × 0.08
2θ range for data collection /°	6.914 to 153.578

Table S4. Crystal data and structure refinement for TTTL.

Empirical formula	$C_{24}H_{23}N_9O_7$
Formula weight	549.51
Temperature/K	193.00
Crystal system	monoclinic
Space group	C2/c
a / Å	40.322 (4)
b / Å	7.0682 (7)
c / Å	17.4724 (18)
α / °	90
β / °	100.180 (3)
γ / °	90
V / Å^3	4901.3 (9)
Z	8
ϱ/(g·cm^{-3})	1.489
μ / mm^{-1}	0.113
Crystal size / mm^3	0.13 × 0.12 × 0.1
2θ range for data collection /°	4.738 to 55.074

5. Degradation of the film during aging

Table S5. Classification of degradation grade of films in UV aging tester.

Empirical formula	0h	48h	96h	144h
PVC	0	0	1	2
PVC/TTTL	0	0	0	0
PVC/TTTJ	0	0	0	0
PVC/TTTD	0	0	0	1
PVC/TTTB	0	0	0	1
PVC/UV-0	0	0	0	1
PVC/UV-327	0	0	0	0

Notes: Grade 0: Grade 1: Cracks or holes < 2 cm; Grade 2: Cracks or holes > 2 cm; Grade 3: Large area fracture; Grade 4: Break into pieces
